# Phage-Based Approaches to Chronic *Pseudomonas aeruginosa* Lung Infection in Cystic Fibrosis

**DOI:** 10.3390/antibiotics15020125

**Published:** 2026-01-27

**Authors:** Wontae Hwang, Ji Hyun Yong, Bryan R. Lenneman, Lael M. Yonker

**Affiliations:** 1 Department of Pediatrics, University of Texas Southwestern Medical Center, Dallas, TX 75390, USA; wontae.hwang@utsouthwestern.edu (W.H.); jihyun.yong@utsouthwestern.edu (J.H.Y.); 2 Mucosal Immunology and Biology Research Center, Division of Pediatric Gastroenterology and Nutrition, Massachusetts General Brigham, Boston, MA 02129, USA; 3 Department of Pediatrics, Harvard Medical School, Boston, MA 02115, USA

**Keywords:** bacteriophage therapy, *Pseudomonas aeruginosa*, cystic fibrosis, bacterial persistence, hypermutator phenotypes, host-phage interactions

## Abstract

Chronic *Pseudomonas aeruginosa* lung infections in cystic fibrosis (CF) represent one of the most treatment-refractory bacterial diseases, sustained by biofilm formation, metabolic dormancy, and adaptive antibiotic resistance evolution. While bacteriophage (phage) therapy has emerged as a promising alternative for multidrug-resistant (MDR) pathogens, clinical studies in CF have demonstrated transient reductions in bacterial burden without achieving complete eradication. This review integrates molecular, evolutionary, and immunological findings to explain the multifactorial barriers that limit phage therapeutic efficacy in chronic CF infections. We highlight three major obstacles: (i) bacterial dormancy and persistence within biofilms that restrict phage adsorption and replication; (ii) hypermutability and extensive genotypic diversification of CF-adapted *P. aeruginosa*, which accelerate phage resistance evolution and necessitate broad host-range coverage; and (iii) CF-specific immune constraints—including a dysfunctional innate immune system and phage-neutralizing humoral immunity—that reduce phage bioavailability and undermine sustained bacterial clearance. Emerging strategies to overcome these challenges include the discovery of dormant-targeting phages capable of replicating in metabolically quiescent cells, evolution-informed phage training to delay resistance evolution, and synthetic phage engineering approaches designed to disrupt biofilms and expand host-range coverage. In parallel, computational or artificial intelligence (AI)-guided frameworks for phage cocktail design and cystic fibrosis transmembrane conductance regulator (CFTR) modulator-mediated restoration of host immune function together offer a more integrated therapeutic paradigm that unites phage biology and host immune context. By unifying clinical outcomes with mechanistic, evolutionary, and immunological perspectives, this review outlines a next-generation framework for phage therapy in CF aimed at achieving more durable therapeutic outcomes.

## 1. Introduction

Cystic fibrosis (CF) is a life-limiting genetic disorder caused by mutations in the cystic fibrosis transmembrane conductance regulator (CFTR) gene, resulting in defective chloride ion transport across epithelial cells [1]. This dysfunction produces abnormally thick mucus in the airways, fostering chronic *Pseudomonas aeruginosa* infections that drive progressive respiratory decline [2,3,4]. Historically, CF lung disease has been managed through aggressive airway clearance, chronic and episodic antibiotic therapy, and supportive respiratory care, which have substantially extended patient survival [5]. However, antibiotic-centered strategies often fail to achieve durable clearance of chronic *P. aeruginosa* colonization, underscoring the need for complementary or alternative antimicrobial approaches [5]. In particular, the rise in multidrug-resistant (MDR) and extensively drug-resistant (XDR) *P. aeruginosa* strains has further complicated CF management, prompting interest in alternative approaches [6,7].

While CFTR modulator therapy (small-molecule treatments that partially restore CFTR function) has dramatically improved airway surface liquid hydration and pulmonary function and reduced pulmonary exacerbations [8], improvement in airway chloride transport does not eradicate *P. aeruginosa* colonization [9]. In fact, the vast majority of people with CF who were colonized with *P. aeruginosa* prior to initiating CFTR modulator therapy remain colonized with pathogenic bacteria [9]. Corresponding with persistent colonization, infected CF airways exhibit increased innate immune activation [10], including neutrophil influx and activation, which drives ongoing airway injury via release of serine proteases and neutrophil extracellular traps. Despite CFTR modulation, nearly 20% of people with CF continue to experience pulmonary exacerbations requiring intravenous antibiotics each year [11]. Thus, while CFTR modulator therapy has revolutionized CF management, airway infection remains a significant problem, especially in the setting of multidrug resistance.

Bacteriophages (phages), viruses that specifically infect and lyse bacteria, have emerged as a promising therapy for MDR/XDR infections due to their high host specificity and ability to self-amplify at infection sites without harming commensal flora [12]. Recent trials and compassionate-use cases in CF have confirmed the safety and transient efficacy of phage therapy but have not achieved complete bacterial eradication [7,13,14]. Together, these findings underscore that phage therapy faces multiple biological and therapeutic constraints across infection settings, with the CF lung representing a particularly challenging environment. In the CF lung, these constraints are amplified by biofilm-associated persister cells that remain metabolically dormant and refractory to lytic phage replication, hypermutable lineages that evolve phage resistance and promote phenotypic diversification, and immune dysfunction that compromises host-phage cooperation required for durable bacterial clearance.

**Figure 1:**
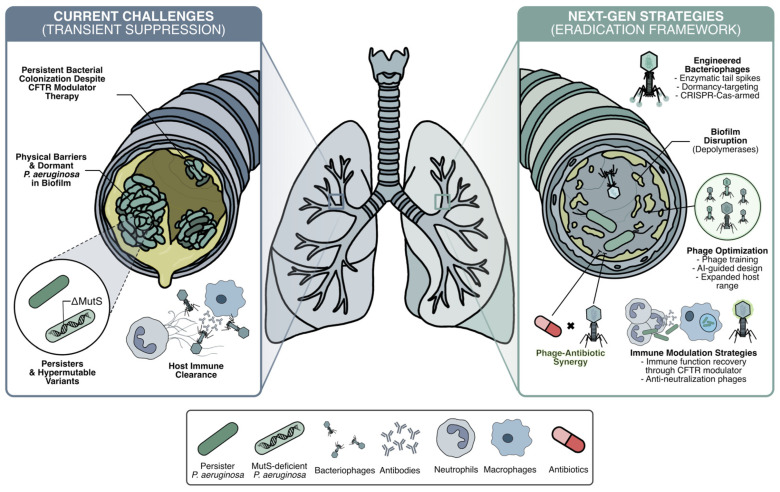
Overview of the major barriers that may limit the efficacy of bacteriophage (phage) therapy against *Pseudomonas aeruginosa* infection in the cystic fibrosis (CF) lung, and emerging strategies to overcome them. (**Left**) Key challenges contributing to transient bacterial suppression during phage therapy in CF airways, including persistent colonization despite cystic fibrosis transmembrane conductance regulator (CFTR) modulator therapy, physical shielding by biofilm architecture, the presence of persister and metabolically dormant subpopulations, hypermutable lineages that accelerate adaptive escape, and CF-specific immune dysfunction (Section 3). (**Right**) Next-generation strategies to improve bacterial control and therapeutic durability. Phage–antibiotic combinations and depolymerase enzymes target biofilm disruption and enhance bacterial accessibility (Section 4.1). Dormancy-targeting phages and CRISPR-Cas-armed engineered phages eliminate dormant and persister populations within biofilms (Section 4.2). Optimization of phage cocktails through evolutionary training, host-range expansion, and artificial intelligence (AI)-guided design broadens strain coverage, minimizes antagonistic interactions, and coordinates selective pressure across heterogeneous bacterial populations (Section 4.3). Host-directed immune modulation, including CFTR modulator therapy and strategies to limit antibody-mediated phage neutralization, may further support sustained antibacterial activity in the CF airway (Section 4.4).

This review synthesizes recent clinical and mechanistic advances that address these challenges, with an emphasis on innovative strategies that integrate evolutionary, immunological, and engineering perspectives (Figure 1). Collectively, these developments provide a framework for advancing phage therapy from transient bacterial suppression toward sustained eradication in chronic CF lung infections.

## 2. Clinical Evidence of Phage Therapy in Cystic Fibrosis

In the United States, phage products intended to treat disease are classified as biological products and are regulated by the Food and Drug Administration (FDA) [15]. Clinical implementation of phage therapy is conducted under the Investigational New Drug (IND) pathway, with individual mechanisms designed to accommodate individual clinical scenarios [16]. The standard IND route represents a classical developmental pathway, requiring Phase I, II, and III clinical trials demonstrating safety and efficacy. In cases of life-threatening conditions requiring emergency treatment, Expanded Access and Single-Patient INDs may be approved for compassionate use [16]. Compassionate use approval requires strong rationale for treatment in the absence of clinically available options, Institutional Regulatory Board (IRB) approval, and patient informed consent [17,18,19]. As part of this approval, the FDA requires detailed information regarding manufacturing processing, certificate of analysis documentation of lytic efficacy against patient’s clinical isolates, endotoxin quantification, microbiological purity testing, and additional safety and manufacturing details [17,18,19]. Because specific phages are often selected to target individual infections, rapid scalability of phage products is limited. This personalized approach has resulted in extensive utilization of compassionate-use approval pathways, although some strategies to commercialize phage cocktails have been developed [18,19].

Across numerous case reports and series, including bacteremia, pneumonia, osteoarticular, and prosthetic infections, phage treatment consistently demonstrated favorable tolerability, with most studies reporting no serious treatment-related adverse events and frequent clinical improvement (Table 1). Notably, in an individual with CF, intravenous (IV) administration of a fixed four-phage cocktail achieved complete resolution of chronic MDR *P. aeruginosa* pneumonia without adverse effects, followed by a sustained infection-free period and successful bilateral lung transplantation [20]. Collectively, these early safety and efficacy signals across both CF and non-CF settings established the feasibility of systemic and local phage delivery, while underscoring the need for controlled, standardized clinical trials in CF to define therapeutic benefit and durability.

Early compassionate-use applications frequently relied on intravenous administration, which enables rapid systemic distribution and is well suited for bacteremia or disseminated infections [12,37]. However, IV-delivered phages are rapidly sequestered and cleared by hepatic and splenic components of the reticuloendothelial system, resulting in short systemic half-lives and variable bioavailability at pulmonary sites [37,38,39]. Systemic exposure increases interaction with humoral immune components, raising concerns regarding the development of neutralizing antibodies that can inactivate circulating phages or accelerate clearance during repeated dosing through antibody–complement cooperation [12,40,41,42,43].

In CF, *P. aeruginosa* infection is predominantly localized within the lung, providing strong rationale for direct respiratory delivery of phage therapy [44]. Inhaled or intratracheal administration enables high phage concentrations within infected airways while minimizing systemic exposure [45,46]. Accordingly, recent and ongoing CF clinical studies have increasingly prioritized aerosolized delivery to maximize pulmonary bioavailability in the context of chronic airway infection.

The SWARM-Pa. trial (phase 1b/2a) was a multicenter, double-blind, placebo-controlled study evaluating the inhaled multi-phage cocktail AP-PA02 in CF patients chronically infected with *P. aeruginosa* [23]. In this dose-escalation study, AP-PA02 was well tolerated with no treatment-related serious adverse events, and pharmacokinetic analyses confirmed efficient lung delivery with minimal systemic exposure. Exploratory microbiological assessments revealed a dose-dependent reduction in sputum *P. aeruginosa* burden, consistent with an exposure–response relationship at higher airway phage concentrations [23,24,25]. Although the study was not powered for efficacy, these findings established the clinical feasibility and safety of repeated inhaled phage administration in CF.

The BX004-A randomized trial (phase 1b/2a) further advanced the evaluation of inhaled phage therapy using a standardized three-phage cocktail delivered via nebulization [13,21]. Treatment was well tolerated in this early-phase study, with no phage-related adverse events reported during the treatment period, and significant but transient reductions in sputum bacterial density compared with placebo [13]. Pharmacokinetics analyses confirmed efficient lower-airway delivery; however, no sustained phage accumulation or durable bacterial eradication was observed, and all patients remained colonized by their original strain throughout treatment [13]. Importantly, no phage- or antibiotic-resistant isolates emerged, and sputum microbiome profiling revealed reduced *P. aeruginosa* relative abundance with preservation of overall community structure, supporting the safety and ecological specificity and short-term safety of this approach [13].

However, in late 2025, the ongoing phase 2b clinical development of the BX004 nebulized cocktail was discontinued following independent safety reviews and internal analyses that identified an unexpectedly high rate of adverse events in the larger, longer-duration trial setting [22]. While earlier phase 1b/2a data from a smaller cohort demonstrated encouraging tolerability, the later trial’s larger sample size, prolonged dosing period, and more comprehensive safety monitoring revealed safety signals not evident in the initial study. This outcome underscores the challenges of translating early safety and microbiological signals into durable clinical benefit in CF and highlights the need for cautious extrapolation when advancing phage therapies into later-phase development [22].

In parallel, a Yale-led personalized inhaled phage therapy program evaluated a compassionate-use, evolution-informed phage selection strategy for CF patients with MDR or pan-drug-resistant (PDR) *P. aeruginosa* lung infections [7]. Patient-specific isolates were screened against environmental phages and combinations of virulent phages targeting distinct bacterial receptors were selected based on predicted evolutionary trade-offs. Nebulized therapy administered daily for 7–10 days was safe and well tolerated, producing rapid reductions in sputum bacterial burden and modest but significant improvement in lung function. Genomic analyses demonstrated that phage-induced receptor mutations frequently occurred in efflux pumps, pili, and lipopolysaccharide (LPS) biosynthesis genes and were associated with reduced virulence or restored antibiotic susceptibility in vivo. In contrast to standardized cocktail approaches, this strategy leveraged personalized phage selection to steer bacterial adaptation toward clinically beneficial phenotypes. Metagenomic profiling revealed no disruption of airway microbial community structure. Whole-genome sequencing confirmed that pre- and post-therapy *P. aeruginosa* isolates remained highly related, indicating persistence of the original colonizing lineages despite transient CFU reductions [7].

## 3. Mechanistic Barriers to Durable Phage Therapy in Chronic CF Infections

Across these studies, a consistent pattern emerges: both standardized phage cocktails and personalized phage strategies produce short-term reductions in *P. aeruginosa* burden but rarely achieve durable eradication in chronic CF lung infections. In the BX004-A trial, genomic analyses and clinical follow-up indicated persistence of the original colonizing lineages during and after dosing [13], while the Yale compassionate-use program documented rapid adaptive responses under phage pressure despite transient clinical improvement [7]. These findings indicate that limited therapeutic durability arises from fundamental biological constraints rather than insufficient phage delivery alone. Such constraints reflect the convergence of bacterial physiological heterogeneity, accelerated evolutionary adaptation, and CF-specific immune dysfunction. Below, we synthesize the mechanistic basis of these barriers and their interactions in the CF airway.

### 3.1. Physiological Persistence and Structural Barriers in Chronic CF Infection

Productive phage infection is tightly constrained by the physiological and structural state of bacterial hosts. In chronic CF airway infections, *P. aeruginosa* populations exhibit pronounced heterogeneity characterized by metabolic dormancy, persister enrichment, envelope remodeling, and biofilm formation, which collectively restrict phage adsorption, replication, and propagation.

Persister cells are transiently dormant phenotypic variants that tolerate antibiotics and other stresses without acquiring genetic resistance [47,48]. Persister formation is promoted by nutrient deprivation, hypoxia, activation of the stringent response (RelA/SpoT), and toxin–antitoxin (TA) systems that suppress translation and induce metabolic dormancy [49,50]. Clinical studies consistently demonstrate enrichment of high-persister (Hip) phenotypes in chronic infections. Early work showed that late-stage CF isolates produced markedly higher levels of drug-tolerant persisters than early isolates, even when antibiotic susceptibility was retained [51]. A large cohort study of 460 isolates from 39 CF patients detected Hip phenotypes in isolates from 56% patients, with Hip status strongly associated with lineage persistence and treatment failure [52].

Phage replication is similarly constrained by host metabolic inactivity. Although diverse phages can adsorb to deep-dormant *Escherichia coli* and *P. aeruginosa*, productive replication is severely impaired under these conditions, with phage propagation resuming only upon host metabolic reactivation [53,54,55,56,57]. Consistent with this, lytic T2 phages can enter *E. coli* persister cells but fail to produce progeny or cause lysis, resulting in more than a 10^5^-fold reduction in phage propagation compared with exponentially growing cells [58]. Under extreme energy limitation, phage adsorption and DNA injection efficiencies are also reduced, supporting a two-step infection model in which phages reversibly bind and initiate productive infection only when sufficient host energy is available [59].

Physiological dormancy is further linked to structural remodeling of the bacterial envelope. Super-resolution microscopy demonstrates nanoscale reorganization of LPS in *P. aeruginosa* and *E. coli* persister cells, a common receptor for many *P. aeruginosa* phages [60]. Such receptor heterogeneity likely diminishes phage binding efficiency. Biofilm architecture imposes an additional spatial barrier, as diffusion-limiting biofilm matrices restrict phage penetration into deeper layers and metabolic gradients constrain productive infection within slow-growing subpopulations [61,62,63,64,65,66]. CF-relevant biofilm models demonstrate that spatial heterogeneity leaves subsets of bacteria effectively shielded from phage exposure, limiting uniform phage–bacteria interactions across the biofilm [63].

Together, physiological dormancy, envelope remodeling, and biofilm-mediated spatial protection form a multilayered barrier that restricts productive phage infection in chronic CF airway disease and likely contributes to persistent bacterial survival following therapy.

### 3.2. Persister-Linked Mutagenesis and Hypermutator-Driven Diversification

Physiological persistence not only protects bacteria from immediate killing but also promotes evolutionary escape from diverse sources of stress. Experimental studies demonstrate that persistence is pleiotropically linked to increased mutation rates, independent of viable cell number [67]. In *E. coli*, high-persistence mutants (*hipA7* and oppB*) exhibit 2.8-fold and 5.0-fold increases in mutation frequency relative to wild type, whereas low-persistence derivatives display reduced mutation frequencies [67]. These findings indicate that persister-rich populations disproportionately seed genetic resistance and adaptive diversification.

In CF, this effect is amplified by the high prevalence of hypermutable *P. aeruginosa* lineages. Defects in DNA mismatch repair genes such as *mutS*, *mutL*, and *uvrD* result in mutation frequencies approximately 100-fold higher than wild type [68,69,70]. In one cohort of 30 CF patients, 36% were chronically colonized by hypermutable strains, most commonly due to *mutS* defects [70]. Longitudinal analyses show that these hypermutator lineages persist for years, diversify extensively across lung niches, and coexist with non-mutator populations to generate substantial intra-host heterogeneity [69,71]. Such diversification manifests phenotypically as variation in motility, quorum sensing, auxotrophy, and antibiotic susceptibility [72,73]. Notably, in vitro biofilm growth alone can generate pronounced heterogeneity, including genotypic variation in LPS biosynthesis genes and corresponding phenotypic diversity in phage susceptibility within a single population [63].

In the context of phage therapy, persisters likely provide a physiological refuge from initial killing, while hypermutable variants act as evolutionary engines that rapidly explore resistance space under sustained selective pressure [71,74]. Together, these interconnected processes help sustain chronic infection, reshape bacterial surface repertoires over time, and complicate durable therapeutic coverage.

### 3.3. CF-Specific Immune Constraints on Phage Therapy

Phage efficacy is further shaped by the host immune environment. In acute respiratory infection models, effective bacterial clearance requires synergy between phages and innate immune effector cells, particularly neutrophils [75]. While phages reduce bacterial burden, neutrophils are essential for eliminating residual and phage-resistant subpopulations, highlighting the importance of host-phage cooperation [75]. This immune–phage synergy, however, is profoundly altered in the CF lung.

Although neutrophils dominate the inflammatory infiltrate in CF lungs, their antimicrobial function is severely dysregulated. CF neutrophils exhibit defective chemotaxis, impaired phagosomal acidification, reduced oxidative killing, and excessive neutrophil extracellular trap formation [76,77]. Loss of CFTR function disrupts chloride transport within neutrophil phagosomes, compromising hypochlorous acid generation and limiting intracellular bacterial killing [77]. As a result, neutrophils predominantly drive chronic inflammation and tissue injury, potentially diminishing immune support for phage-mediated bacterial control rather than facilitating effective pathogen clearance.

Macrophages further constrain therapy by limiting phage bioavailability in the lung. In vivo studies demonstrate that alveolar macrophages phagocytose phages, reducing local phage density and accelerating clearance [78]. Depletion of alveolar macrophages enhances phage-mediated bacterial clearance, despite worsening infection outcomes in the absence of phage treatment, underscoring a context-dependent trade-off between immune protection and therapeutic efficacy [78]. In CF, macrophage function is broadly dysregulated. CF macrophages exhibit impaired bacterial phagocytosis and killing, altered expression of pattern-recognition receptors, and exaggerated yet functionally ineffective inflammatory responses, including during early disease stages and prior to the establishment of chronic infection [79,80,81,82,83]. CFTR-dependent defects also drive transcriptional reprogramming in macrophages, including dysregulated interferon signaling and delayed resolution of inflammation [83,84,85].

Humoral immunity represents an additional constraint. Neutralizing anti-phage antibodies emerge in approximately 38% of patients receiving invasive (intravenous or intralesional) phage therapy, typically within 6–35 days of initiation. In contrast, such responses were not detected in patients treated exclusively via topical or nebulized routes [14]. However, recent reports indicate that neutralizing anti-phage antibodies can also arise following nebulized therapy in CF [86]. In the CF lung, phage efficacy may be limited by innate immune dysfunction that reduces immune-assisted bacterial clearance and by therapy-induced neutralizing antibodies that can directly inhibit phage activity, potentially contributing to the transient nature of therapeutic responses observed clinically.

## 4. Overcoming Barriers to Phage Therapy in Chronic CF Infection

The preceding section illustrates how biofilm structure, bacterial persistence, accelerated evolutionary adaptation, and CF-specific immune dysfunction may limit the durability of phage therapy in chronic *P. aeruginosa* lung infection. In the following section, we consider emerging strategies that could help mitigate these constraints.

### 4.1. Phage–Antibiotic Synergy and Depolymerase-Based Strategies for Biofilm Disruption

#### 4.1.1. Phage–Antibiotic Synergy in Biofilm Eradication

Biofilm formation in chronic *P. aeruginosa* infections restricts the penetration and efficacy of both antibiotics and phages [87,88,89,90]. Accumulating evidence indicates that phage–antibiotic combinations can synergistically disrupt biofilms and enhance bacterial killing [91,92]. Large-scale analyses demonstrate that specific phage–antibiotic pairs exhibit reproducible synergy across diverse clinical isolates, including under biofilm growth conditions [91]. Consistent with these findings, dynamic biofilm models showed that combined phage–ciprofloxacin or phage–meropenem regimens achieved approximately 4–5 log_10_ reductions in biofilm-associated bacterial burden while reducing the emergence of both phage and antibiotic resistance compared with monotherapies [92].

Beyond pharmacodynamic synergy, the evolutionary independence of phage and antibiotic resistance mechanisms further limits the likelihood of simultaneous resistance emergence [93]. Moreover, phage resistance often carries genetic tradeoffs such as modifications in outer membrane or efflux pump function that reduce virulence or restore antibiotic susceptibility, reinforcing the therapeutic benefit of combined approaches [94,95,96]. However, phage–antibiotic combinations are not universally synergistic. Certain antibiotic classes or dosing regimens may interfere with phage replication, and high antibiotic concentrations can suppress phage propagation [95,97]. Accordingly, careful optimization of antibiotic selection and dosing remains essential to maximize therapeutic benefit while minimizing antagonistic interactions.

#### 4.1.2. Depolymerase-Mediated Biofilm Disruption

Another complementary approach to biofilm control relies on enzymatic degradation of the extracellular polymeric substances that maintain biofilm integrity. Phage-encoded depolymerases enable targeted disruption of the biofilm matrix and enhanced bacterial accessibility. Examples include the O-specific polysaccharide lyase encoded by phage LKA1, which cleaves B-band LPS [98], alginate lyases derived from CF-associated phages that depolymerize alginic acid capsules [99], and depolymerases from the lytic phage IME180 that degrade exopolysaccharides and disrupt established biofilms [100]. By degrading matrix components, these enzymes may increase permeability and promote bacterial exposure to both immune defenses and therapeutic agents.

In parallel, bacterially encoded matrix-degrading enzymes can similarly potentiate biofilm disruption. Exogenous application of glycoside hydrolases such as PslG and PelA, or alginate lyases derived from environmental bacteria, selectively cleave Psl/Pel or alginate exopolysaccharides, leading to reduced biofilm biomass, enhanced antibiotic efficacy, and improved access of innate immune cells [101,102,103,104]. Collectively, these studies highlight depolymerase-mediated matrix degradation as a convergent mechanism to overcome structural barriers in biofilm-associated infections. Whether encoded within phages or supplied as enzymatic adjuvants, depolymerase-based strategies offer a mechanistically grounded approach to improving biofilm clearance in chronic *P. aeruginosa* infections.

### 4.2. Phage-Based Strategies to Target Bacterial Persistence

#### 4.2.1. Anti-Persister Adjuvants as Phage Therapy Complements

Combining phages with anti-persister agents offers a complementary strategy to reduce dormant, treatment-refractory cells. Lytic infection itself can transiently induce persister formation, generating metabolically inactive survivors that evade both phage and antibiotic killing [58,105]. Co-administration of phages with anti-persister compounds may therefore help limit bacterial rebound following treatment.

Among candidate agents, eravacycline accumulates within *P. aeruginosa* biofilms and retains activity against persister populations during resuscitation [106]. In a murine lung infection models, exposure of pre-formed persisters to eravacycline enhanced bacterial clearance, suggesting that intracellularly retained drug can act during early resuscitation in vivo [106]. Eravacycline also remained effective against persisters induced by multiple stressors and exhibits improved activity when combined with antibiotics such as ceftazidime and tobramycin [106]. Mitomycin C, a DNA cross-linking agent, kills persisters through a growth-independent mechanism and shows activity against biofilm-associated bacteria across experimental and animal models [107]. However, its clinical translation is limited by systemic toxicity and tissue-damage concerns, and its antibiofilm effect primarily reflects bactericidal killing rather than genuine biofilm dispersal [107].

#### 4.2.2. Dormancy-Targeting Phages

Despite dormancy imposing a major barrier to phage replication [53,54,57], recent studies demonstrate that some phages have evolved to overcome this limitation [53]. Phage Paride was identified as the first known *P. aeruginosa* phage capable of replicating directly within deep-dormant, antibiotic-tolerant bacteria [53]. Unlike most virulent phages that enter hibernation upon encountering non-growing hosts, Paride hijacks bacterial stress-response pathways, particularly the (p)ppGpp stringent response and the stationary-phase sigma factor RpoS, to activate its replication program [53]. Genetic disruption of *relA*/*spoT* or *rpoS* abolished Paride replication in dormant bacteria while preserving infection of actively growing bacteria, demonstrating that Paride exploits dormancy-associated regulatory circuits to initiate replication [53].

Although this approach remains at an early, preclinical stage, Paride’s therapeutic potential is further underscored by its synergy with antibiotics. While Paride alone eliminated approximately 99% of dormant bacteria and meropenem alone was ineffective, their combination completely sterilized deep-dormant *P. aeruginosa* cultures in vitro and produced ~3-log_10_ reductions in bacterial burden in a murine tissue-cage model of chronic infection [53]. The synergy is proposed to arise from a “forced resuscitation mechanism”, in which phage-mediated lysis releases nutrients or signals that awaken neighboring persisters, rendering them susceptible to β-lactam killing [53]. These findings reframe bacterial dormancy as a dynamic and therapeutically exploitable state rather than a passive form of drug tolerance.

#### 4.2.3. CRISPR-Cas-Armed Phages

Complementing naturally evolved dormancy-targeting phages, clustered regularly interspaced palindromic repeat associated protein (CRISPR-Cas)-armed phages represent a synthetic approach to eliminate bacterial subpopulations that escape conventional lytic infection, which has thus far been evaluated exclusively in experimental systems [108]. In this strategy, phages deliver programmable CRISPR payloads that induce sequence-specific DNA cleavage upon infection, reducing bacterial survival even when phage replication is inefficient [108]. Experimental studies demonstrate that CRISPR-equipped phages suppress the emergence of phage-tolerant mutants and outperform wild type phages by disabling receptor-escape variants, including under growth-restricted or biofilm-like conditions [108].

Beyond direct killing, phage therapy can also take advantage of predictable evolutionary trade-offs that accompany bacterial escape from phage predation. A classic example is phage OMKO1, which targets the OprM component of the MexAB/MexXY efflux system [94]. Resistance mutations disrupt efflux activity and restore antibiotic susceptibility, coupling phage resistance to a clinically favorable loss of multiple resistance [94]. Similar trade-offs have been reported in evolution-trained phage cocktails, where resistance-associated mutations impair O-antigen synthesis, reduce biofilm formation, and decrease bacterial fitness [96]. Building on this principle, CRISPR-Cas-armed phages offer a means to deliberately extend such trade-offs by targeting essential or functionally constrained genomic regions [109,110,111]. Although resistance may still arise through alterations in CRISPR-associated or guide RNA loci [110,112], these adaptations typically incur additional fitness costs and rarely eliminate selective pressure entirely.

Collectively, anti-persister adjuvants, dormancy-targeting phages, and CRISPR-Cas-armed phages outline an integrated, physiology-informed strategy to address bacterial dormancy across both actively replicating and metabolically quiescent populations.

### 4.3. Phage Cocktail Optimization

Phage cocktails represent a rational strategy to overcome the limitations of single-phage therapy, including narrow host range, rapid resistance emergence, and biofilm-associated heterogeneity. By combining phages that target distinct bacterial receptors, cocktails achieve broader coverage and enhanced efficacy [91,96,113]. In vitro studies demonstrate that non-redundant phage cocktails produce deeper and more sustained killing of *P. aeruginosa* biofilms, including those formed by XDR clinical isolates, while reducing regrowth associated with receptor modification or resistance [96,113]. Reflecting these advantages, early clinical trials increasingly employ multi-phage formulations rather than single phages [7,13].

#### 4.3.1. Ecological Interactions and Resistance Dynamics

Despite these advantages, phage mixtures are not inherently synergistic, and ecological interactions among phages can strongly influence therapeutic efficacy. An experimental study shows that diverse lytic phages can coexist on a single clonal host due to bacterial phenotypic heterogeneity [114]. However, most pairwise interactions are antagonistic or amensalistic rather than cooperative [114]. Such antagonism can arise from competition for overlapping host subpopulations, mismatched replication timing, or adsorption interference, occasionally resulting in competitive exclusion.

**Figure 2:**
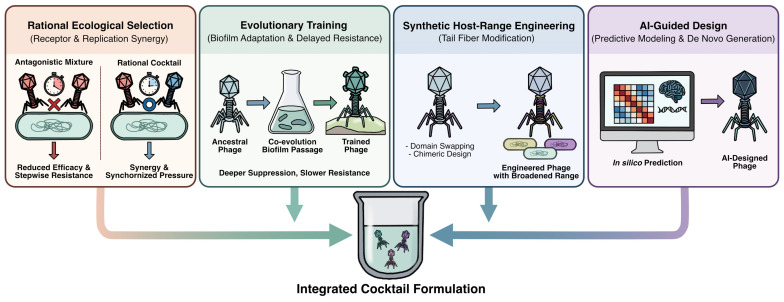
Integrated strategies for optimizing phage cocktails against *P. aeruginosa* in CF. Cocktails are rationally designed using four key pillars (from (**left**) to (**right**)): (1) ecological selection for receptor diversity and replication synchrony to prevent antagonism; (2) evolutionary training to enhance biofilm penetration and delay resistance; (3) synthetic engineering of tail fibers to broaden host-range coverage; and (4) AI-guided modeling for predictive design and de novo phage generation. These combined approaches shift phage therapy from empirical mixtures toward engineered systems optimized for sustained bacterial suppression in the CF lung.

Population-level evolutionary dynamics further constrain cocktail efficacy. Modeling studies demonstrate that resistance to multi-phage cocktails can evolve through stepwise adaptation when individual phages impose selective pressure asynchronously, reflecting variability in replication kinetics and burst sizes [115]. Only a small proportion (~9%) of randomly paired phages exert sufficiently synchronized selective pressure to robustly suppress resistance, and the probability of resistance prevention increases roughly linearly, rather than exponentially, with cocktail size [115]. These findings underscore the importance of rational phage cocktail design incorporating ecological compatibility to minimize antagonistic interactions and replication synchrony to limit sequential resistance selection (Figure 2).

#### 4.3.2. Evolutionary Optimization Through Phage Training

To mitigate resistance emergence, coevolutionary “phage training” approaches pre-adapt phages against anticipated bacterial defenses. In controlled co-culture experiments, trained phages evolved through extended propagation with their hosts achieved substantially deeper and more sustained bacterial suppression than their ancestral counterparts, with delayed resistance emergence and reduced mutation frequencies [116]. Directed evolution within biofilm environments further enhances phage efficacy by selecting variants with improved suppression of spatially structured bacterial populations [63,96]. Together, these approaches highlight that pre-adapting phages through evolutionary training under infection-relevant conditions can enhance phage persistence and efficacy in complex infection settings.

#### 4.3.3. Synthetic Expansion of Host Range

Synthetic host-range engineering directly broadens the bacterial subsets that can be targeted [117]. Structure-guided diversification of phage tail-fiber receptor-binding domains enables engineered phages to infect resistant mutants while preserving stability and fitness [118]. Rational recombination of receptor-binding domains with complementary specificities further expands host range without extensive mutagenesis [108]. Advances in structural biology have enabled predictable and tunable reprogramming of phage host specificity through modular domain swapping [119]. In addition, metagenomic mining and deep mutational scanning have identified conserved motifs that can be transplanted across phylogenetically distant phages, producing variants with novel adsorption profiles inaccessible through natural evolution alone [120]. These strategies offer a modular framework for host-range expansion with increasing mechanistic precision.

#### 4.3.4. AI-Guided Design of Next-Generation Phage Cocktails

As cocktail complexity increases, identifying phage combinations that balance receptor diversity, ecological compatibility, and synchronized selective pressure becomes difficult to scale experimentally. Computational and artificial intelligence (AI)-guided approaches have emerged to address this challenge. Machine learning frameworks predict phage–host specificity from genomic features, including receptor-binding proteins and host surface determinants, enabling strain-level matching even across low sequence-similarity regimes [121,122,123,124,125,126].

However, predictive accuracy remains limited by sparse and unevenly curated training data [127], and current models tend to emphasize receptor-level interactions while underrepresenting downstream cellular and evolutionary processes [127]. Despite these challenges, computational selection of complementary phages has already demonstrated practical value in identifying effective combinations against previously untested clinical isolates [128]. More recently, genome-scale language models have generated viable, AI-designed phage genomes with defined lytic properties and host tropism, including variants capable of overcoming resistance to naturally occurring phages [129]. Collectively, these approaches represent a still predominantly experimental but rapidly advancing frontier in phage therapeutic design.

### 4.4. Host Immune Modulation as an Adjunct to Phage Therapy in Cystic Fibrosis

Emerging evidence indicates that CFTR modulator therapy partially restores innate immune function in CF. Highly effective triple-combination therapy (elexacaftor/tezacaftor/ivacaftor; ETI) improves neutrophil antimicrobial capacity and attenuates excessive inflammatory signaling, consistent with restored CFTR-dependent ion flux [130,131]. In macrophages, CFTR modulators enhance CFTR expression and plasma-membrane localization, improve phagocytosis and intracellular killing, and promote efferocytosis of apoptotic neutrophils [132]. CFTR modulation also reverses intrinsic immune dysregulation at the transcriptional level, including impaired type I interferon signaling, supporting a cell-intrinsic role for CFTR in macrophage immune programming [83].

Despite these improvements, immune recovery remains incomplete and heterogeneous. A subset of patients exhibit persistent neutrophil activation linked to dysregulated calcium signaling and ongoing degranulation [133]. Likewise, macrophage functional recovery shows substantial inter-individual variability [132]. Spatially resolved analyses reveal persistent *P. aeruginosa* infection and neutrophil-dominated inflammation in some patients despite robust systemic CFTR correction [134].

**Figure 3:**
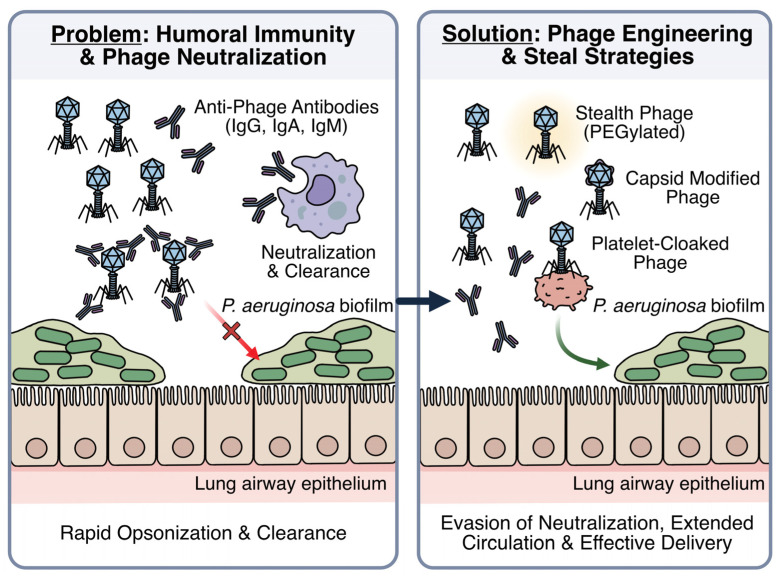
Host immune responses and phage engineering strategies in the CF airway. While CFTR modulator therapies (e.g., ETI) partially restore CFTR function and improve cellular immune mechanisms, humoral immunity poses a significant barrier to therapeutic success. Unmodified phages are rapidly opsonized by anti-phage antibodies (IgG, IgA, IgM), leading to neutralization and premature clearance before they can reach the target *P. aeruginosa* biofilm (**left**). To mitigate these humoral barriers, phage engineering strategies (**right**) are employed, including “stealth” modifications like PEGylation, capsid alterations, or platelet cloaking, which enable phages to evade antibody neutralization, extend circulation time, and achieve effective delivery to the infection site.

Humoral immunity further influences phage pharmacology (Figure 3). Circulating phages are rapidly opsonized and variably neutralized by patient-specific antibody repertoires [135,136]. Anti-phage IgG, IgM, and IgA are commonly detectable at baseline and often increase during therapy, accelerating clearance while showing inconsistent relationships with clinical outcomes, particularly during local administration [136,137,138]. Strategies to reduce immune-mediated phage clearance include selecting phages with low baseline immunogenicity, optimizing delivery routes, and engineering ‘stealth’ phages through capsid modification, glycosylation, PEGylation, or platelet-membrane cloaking [135,137,139,140]. A nuanced understanding of patient-specific immune phenotypes, particularly in the context of CFTR modulator therapy, together with strategies that minimize or evade humoral neutralization, will be essential to achieving more sustained and predictable phage efficacy in the CF airway.

## 5. Summary

Phage therapy is entering a new phase of translational maturity for chronic *P. aeruginosa* infections in CF, evolving from individualized compassionate-use applications toward rigorously designed clinical evaluation. Across early trials and real-world programs, phage treatment has demonstrated a favorable safety profile and reproducible short-term reductions in bacterial burden. Yet, durable eradication of *P. aeruginosa* from the CF airway remains uncommon, emphasizing the intrinsic challenges of chronic airway infection.

As synthesized in this review, limited therapeutic durability arises from the convergence of multiple reinforcing barriers rather than a single dominant failure mode. Dormant and biofilm-embedded bacterial subpopulations restrict phage adsorption and replication, while hypermutable and persister-enriched lineages accelerate adaptive escape under sustained selective pressure. At the same time, CF-specific immune dysfunction, particularly impaired regulation of neutrophils, further constrains sustained phage efficacy. In addition, humoral immune responses elicited during therapy, including the formation of neutralizing antibodies, can accelerate phage clearance and add another layer of variability to treatment outcomes. Together, these physiological, evolutionary, and immunological constraints define a therapeutic landscape in which conventional lytic phages alone are unlikely to achieve durable bacterial control.

Recent advances increasingly reframe these limitations as opportunities for design rather than insurmountable barriers. Dormancy-targeting phages such as Paride demonstrate that bacterial quiescence can be therapeutically leveraged, and co-evolutionary training or directed evolution can generate pre-adapted phages that sustain bacterial suppression while often imposing resistance-associated fitness costs. In parallel, synthetic engineering strategies, including depolymerase-armed, host-range-expanded, and CRISPR-Cas-armed phages, enable more precise targeting of biofilm structure, heterogenous bacterial populations, and genetic resilience. As phage cocktails grow in complexity, computational and AI-guided design frameworks are emerging as important tools for navigating phage–host interactions and optimizing cocktail composition. CFTR modulators, together with circulation-prolonged or immunologically “camouflaged” phages, may help mitigate innate immune dysfunction and reduce immune-mediated clearance of therapeutic phages in the CF airway.

Taken together, these findings suggest that durable phage therapy for CF-associated *P. aeruginosa* infections will require an integrated, systems-level approach that aligns bacterial physiology, evolutionary dynamics, host immunity, and computationally informed phage design. Such integration holds promise to advance phage therapy from a transient adjunct toward a more durable and resilient treatment strategy for chronic CF lung infections.

## Figures and Tables

**Table 1:** Clinical studies and compassionate-use applications of bacteriophage therapy targeting *P. aeruginosa*.

Trial/Case	Bacteria	Infection Type (Cohort)	Phage Treatment	Delivery Method	Dosing/Duration	Safety	Efficacy	Reference
Case report	MDR *P. aeruginosa*	Chronic lung infection (CF adults)	Fixed 4-phage cocktail (AB-PA01)	Intravenous	Every 6 h for 8 weeks	Well tolerated; no treatment-related serious adverse events observed	Resolution of MDR *P. aeruginosa* pneumonia; no recurrence or CF exacerbation within 100 days post-therapy; subsequent successful lung transplantation	[20]
BX004 (Phase 1b/2a)	*P. aeruginosa*	Chronic lung infection (CF adults)	3-phage cocktail (BX004-A)	Inhaled (nebulized)	7 days total: placebo on Day 1; once-daily dose escalation on Days 2–3 (1.4 × 10^8^ → 1.4 × 10^10^ PFU); twice-daily dosing on Days 4–7	Well tolerated; no treatment-related serious adverse events observed	Significant but transient reduction in sputum bacterial density	[13,21]
BX004 (Phase 2b)	*P. aeruginosa*	Chronic lung infection (CF adults)	BX004 cocktail	Inhaled (nebulized)	Planned 8-week twice-daily dosing; trial discontinued before completion	Study discontinued following safety review due to higher-than-expected adverse events; no definitive drug-related toxicity was established	Not reported; earlier Phase 1b/2a showed transient bacterial load reduction	[22]
SWARM-Pa (Phase 1b/2a)	*P. aeruginosa*	Chronic lung infection (CF adults)	AP-PA02 cocktail	Inhaled (nebulized)	Dose-escalation design (single and multiple ascending doses)	Well tolerated; no treatment-related serious adverse events observed	Dose-dependent reduction trend in sputum *P. aeruginosa* burden, with exposure–response relationship at higher phage exposure	[23,24,25]
Personalized program (Yale compassionate-use cohort)	MDR/PDR *P. aeruginosa*	Chronic lung infection (CF adults)	Personalized, evolution-informed phages (OMKO1, TIVP-H6, LPS-5)	Inhaled (nebulized)	7–10 days; once or twice daily; 1 × 10^10^ PFU total per treatment; cocktails (2–3 phages, n = 6) or single phage (n = 3)	Well tolerated; no treatment-related serious adverse events observed; mild transient fever or fatigue reported in some patients	Median 10^4^-fold reduction in sputum bacterial burden; sustained reduction up to day 42; median 6% (mean 8%) improvement in ppFEV_1_	[7]
WRAIR-PAM-CF1 (Phase 1b/2)	*P. aeruginosa*	Chronic lung infection (CF adults)	4-phage mixture (PaWRA01Phi11, PaWRA01Phi39, PaWRA02Phi83, PaWRA02Phi87)	Intravenous	Single-dose escalation: 4 × 10^7^, 4 × 10^8^, or 4 × 10^9^ PFU	Ongoing; safety outcomes not yet reported	Ongoing; no results reported	[26,27]
Tailwind (Phase 2)	*P. aeruginosa*	Non-CF bronchiectasis with chronic lung infection	AP-PA02 cocktail	Inhaled (nebulized)	Twice daily for 10 days	Well tolerated overall; one possibly treatment-related serious pulmonary event	Post hoc intention-to-treat analysis showed reduction in sputum bacterial load; approximately one-third of treated participants achieved ≥ 2-log CFU reduction	[25,28]
Case report	MDR *P. aeruginosa*	Recurrent pneumonia after bilateral lung transplantation	Sequential phage cocktails (AB-PA01, AB-PA01m1, Navy Cocktails 1 & 2)	Intravenous + inhaled	Not reported	Well tolerated; no treatment-related adverse events observed	Resolution of pneumonia; evidence of intrapulmonary phage replication and improved respiratory function	[29]
Case report	MDR *P. aeruginosa*	Refractory bacteremia/sepsis (pediatric patient with congenital heart disease)	2-phage cocktail from U.S. Navy phage library	Intravenous	3.5 × 10^5^ PFU every 6 h; initial 36-h course, resumed after 11 days	Transient clinical decompensation after early doses (initially suspected anaphylaxis, later attributed to cardiac failure); otherwise well tolerated	Blood cultures sterilized within 4–5 days; transient infection control	[30]
Case report	Colistin-only-susceptible *P. aeruginosa*	Septicemia with acute kidney injury (adult)	2-phage cocktail (BFC-1)	Intravenous + local (wound irrigation)	IV infusion (6 h daily) plus wound irrigation every 8 h for 10 days	Well tolerated; no treatment-related serious adverse events observed	Rapid clearance of bacteremia; normalization of inflammatory markers; clinical recovery	[31]
Case series	MDR/XDR *P. aeruginosa*	Severe and chronic infections (Bone, lung, wound, vascular)	Single lytic phage PASA16	Intravenous ± local (wound or joint instillation)	Once or twice daily for 8–49 days (median 14 days)	Well tolerated; mild and reversible adverse events reported	Clinical recovery or remission in 13/15 evaluable patients (86.6%)	[32]
Case report	XDR *P. aeruginosa*	Complex osteoarticular infection (sacroiliac osteomyelitis)	Personalized 4-phage cocktail	Local instillation	Every 3 days over ~14 days	Well tolerated; no treatment-related adverse events observed	Marked local improvement and infection control; patient later died from cancer progression	[33]
Case report	*P. aeruginosa*	Prosthetic aortic graft infection	2-phage cocktail (PP1450, PP1777)	Intravenous	10^10^ PFU of each phage every 12 h for 7 days	Well tolerated; no treatment-related adverse events observed	Clearance of infection with normalization of inflammatory markers and no relapse at 12 months	[34]
Case report	MDR *P. aeruginosa*	Prosthetic vascular graft infection	Tailored 3-phage cocktail (PT07, 14/01, PNM)	Intravenous	7 days: 70 mL/day (10^7^ PFU/mL) infused over 6 h for 3 inpatient days plus 4 outpatient days	Well tolerated; no treatment-related adverse events observed	Clinical and microbiological failure; recurrence associated with increased biofilm formation and phage resistance	[35]
Case report	MDR *P. aeruginosa*	Chronic lung infection (Kartagener syndrome)	Single lytic phage vFB297	Inhaled (nebulized)	First course: 5 × 10^9^ PFU daily × 5 days + 2 booster doses after 2 days; Second course (after relapse): 5 × 10^9^ PFU daily × 5 days	Well tolerated; no treatment-related serious adverse events observed; mild transient drop in O_2_ saturation, transient C-reactive protein rise, single fever event	Reduced sputum bacterial load; computed tomography evidence of progressive partial lung clearance; in vivo evidence of phage replication in sputum samples; clinical relapses in the context of a persisting clonal hypermutator *P. aeruginosa* population.	[36]

Multidrug-resistant (MDR); Extensively drug-resistant (XDR); Pan-drug-resistant (PDR); Plaque-forming unit (PFU); Colony-forming unit (CFU).

## Data Availability

No new data were created or analyzed in this study. Data sharing is not applicable to this article.
